# Advancing Aquatic Ecotoxicology Testing of Anticancer Drugs Through Mesocosms

**DOI:** 10.3390/molecules30244787

**Published:** 2025-12-15

**Authors:** Andrea Carboni, Matteo Calvaresi

**Affiliations:** 1 Dipartimento di Chimica “Giacomo Ciamician”, Alma Mater Studiorum—Università di Bologna, Via Piero Gobetti, 85, 40129 Bologna, Italy; 2 IRCCS Azienda Ospedaliero—Universitaria di Bologna, Preclinical & Translational Research in Oncology Lab (PRO), 40138 Bologna, Italy

**Keywords:** chemotherapeutics, ecotoxicity, emerging contaminants, aquatic ecosystems, environmental impact

## Abstract

The widespread use of anticancer drugs (ACDs) in human therapies determines the occurrence of these potent cytotoxic chemicals into aquatic ecosystems. Nowadays, ACDs are ubiquitous contaminants in wastewater effluents and freshwater compartments, raising urgent questions about their environmental impact. Designed to disrupt cellular proliferation, these compounds are inherently bioactive and can exert toxic effects on non-target organisms even at trace concentrations. Conventional fate and toxicity tests provide important initial data but are limited in ecological realism, often focusing on single-specie and single-endpoint under controlled conditions and overlooking complex interactions, trophic dynamics, and long-term chronic exposures. Knowledge of all these aspects is needed for proper monitoring, assessment, and regulation of ACDs. Simulated ecosystem experiments, such as mesocosms, provide intermediate-scale, semi-controlled platforms for investigating real-world exposure scenarios, assessing ACD fate, and identifying both direct and indirect ecological effects. They offer distinct advantages for evaluating the chronic toxicity of persistent pollutants by enabling realistic long-term contamination simulations and supporting the simultaneous collection of comprehensive hazard and exposure endpoints. This perspective underscores the growing concern surrounding the contamination of ACDs, examines the limitations of traditional assessment approaches, and advocates for mesocosm-based studies as a critical bridge between laboratory research and ecosystem-level understanding. By integrating mesocosm experiments into environmental fate and risk evaluation, we can better predict the behavior and ecological consequences of anticancer pharmaceuticals, guiding strategies to mitigate their impact on aquatic life.

## 1. Introduction

The widespread use of anticancer drugs (ACDs) represents one of the most remarkable achievements of modern pharmacotherapy, substantially contributing to increased survival rates and improved quality of life for cancer patients worldwide. However, their extensive use necessarily implies their release into the environment, which raises concerns about potential negative effects on the ecosystem.

Today, pharmaceutical contamination in freshwater systems represents a global environmental threat [1], and anticancer drugs can be considered contaminants of emerging concern (CECs) because detected at concentrations higher than expected or whose human and environmental risk is yet to be fully understood [2].

**Figure 1:**
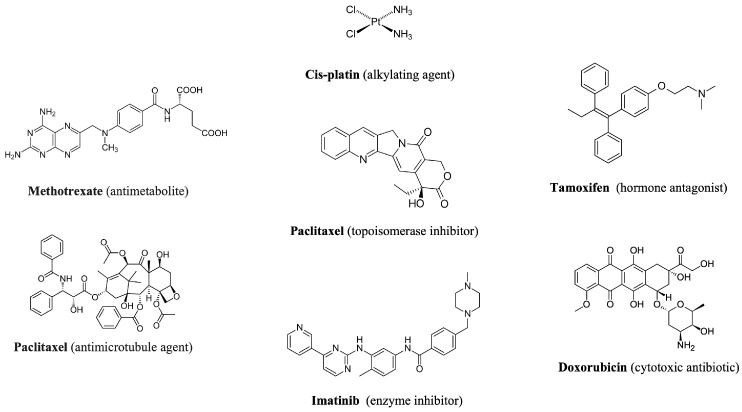
Chemical structure and mechanism of action of representative anticancer drugs.

ACDs encompass a chemically diverse group of compounds, such as alkylating agents, antimetabolites, anti-microtubule agents, cytotoxic antibiotics, protein inhibitors, and hormonal antagonists [3] (Figure 1). Many of these agents are characterized by high biological activity, low biodegradability, and structural stability, which together confer a strong potential for environmental persistence and risk [4]. Designed to disrupt fundamental cellular mechanisms such as DNA replication, mitotic spindle formation, and signal transduction pathways, these compounds inherently possess mutagenic, genotoxic, and cytotoxic properties [5,6].

Their physicochemical persistence, coupled with incomplete metabolism in humans, leads to the excretion of both parent compounds and active metabolites into natural water bodies and wastewater systems [7,8]. In turn, the incomplete removal or degradation in wastewater treatment plants (WWTPs) can result in the continuous discharge of such potent micropollutants into surface waters and, ultimately, the broader environment [9]. Once released, anticancer drugs and their transformation products can persist in aquatic environments at concentrations ranging from the low nanogram to microgram per liter scale [10]. Although these concentrations are typically below ecotoxic thresholds, ACDs showed to induce genotoxic and cytostatic effects in non-target species, including algae, invertebrates, and fish [11,12]. The chronic exposure of aquatic organisms to bioactive agents capable of interfering with cell proliferation, DNA integrity, and oxidative balance raises significant ecotoxicological concerns [13].

The occurrence and ecotoxicity of ACDs have been recently reviewed by several studies [10,11,12,13,14], which concluded that the current knowledge on their fate (e.g., occurrence, persistence, degradation, transformation, environmental partitioning) and effects (e.g., toxicity, bioaccumulation, trophic transfer) is extremely limited and mostly based on standard single-endpoints and single species test [10,11,12,13,14]. Here, we provide a brief overview on these topics and focus on (i) highlighting the current knowledge gaps, especially concerning the realistic ecological assessment of ACDs in a lifecycle perspective and (ii) discussing the use of experimental systems capable of representing a higher ecological complexity, such as mesocosms, in order to overcome many of these limitations and contribute to the evaluation of ACDs environmental fate and impact.

## 2. Occurrence and Impact of ACDs in Freshwater in a Life Cycle Perspective

**Figure 2:**
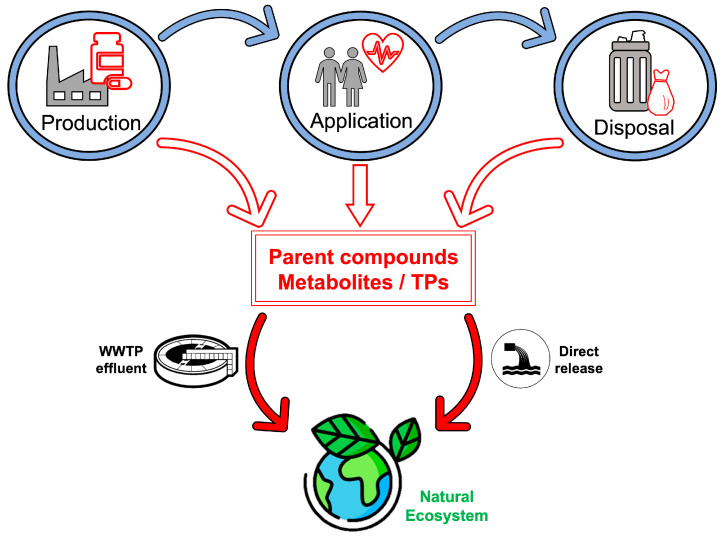
The main pathways for anticancer drugs entrance in the environment. Parent compounds, metabolites, and transformation products (TPs) can occur during production, use, and disposal phases. Natural ecosystem exposure can result following the direct release of such species or due to incomplete removal in wastewater treatment plants (WWTPs).

Defining ACDs occurrence in the environment requires an investigation of the potential release pathways along their lifecycle. As shown in Figure 2, the main pathways can be identified during the production, use, and disposal of the commercialized ADCs.

The release of ACDs during synthesis and production stages likely concerns intact active pharmaceutical ingredients from pharmaceutical industry or research centers. In principle, such an input should be limited, given the high control at manufacturing facilities/research laboratories and further mitigated by downstream sewage water treatment. Nonetheless, wastewater effluents from pharmaceutical manufacturing plants engaged in the synthesis of anticancer agents frequently contain elevated concentrations of these substances [13,15]. Overall, pharmaceutical products in WWTP effluents connected to pharmaceutical structures can be up to 1000 times higher than average WWTPs, and the freshwater compartments represent the first step in environmental hazard after the release of such effluents [16].

A main release pathway for ACDs parental compounds and metabolites is obviously identified during the use phase, i.e., from patients undergoing cancer therapies. Anticancer pharmaceuticals exhibit low metabolic transformation rates and are excreted in their unmetabolized form following the renal or hepatobiliary processing. Since chemotherapy is administered both within hospital settings and through outpatient regimens, effluents originating from medical facilities as well as domestic wastewater have been identified as major conduits for the discharge of these compounds into aquatic environments [8]. In addition, environmental release may also occur incidentally or during end-of-use and end-of-life stages, e.g., by disposal of expired products.

Conventional wastewater treatment plants (WWTPs) are not necessarily engineered to remove such potent micropollutants, resulting in polluted effluents reaching the freshwater compartment [17]. Moreover, ACD degradation and transformation in water matrices can result in the generation of by-product and transformation products (TPs) that may be more toxic, persistent, and less biodegradable than their predecessors [18]. The direct emission of untreated sewage in the environment is another important route in a world where the majority of wastewater is released without being treated [19]. For instance, in southeast Europe, up to 25% of urban wastewater is directly discharged into freshwater systems without any treatment [17], contributing to the continental river’s degradation, which meet good ecological status in only 42% of cases [20].

In summary, aquatic ecosystems are exposed to a mixture of ACD parental compounds, metabolites, and additional transformation products. Therefore, these chemicals are currently detected in WWTP effluents and water bodies worldwide at concentrations ranging from the low ppt to hundreds ppm [14]. Such a ubiquitous presence is a cause for concern due to its potential negative effects on humans and the ecosystem.

ACDs are designed to be cytotoxic, i.e., toxic to cancer cells, with a mechanism of action that often involves interfering with DNA replication and cell growth. Although beneficial for treating cancer, they are not specific to tumor cells, and their use also alters the functions of normal cells, both eukaryote and prokaryote, affecting humans and non-target organisms in the environment.

**Figure 3:**
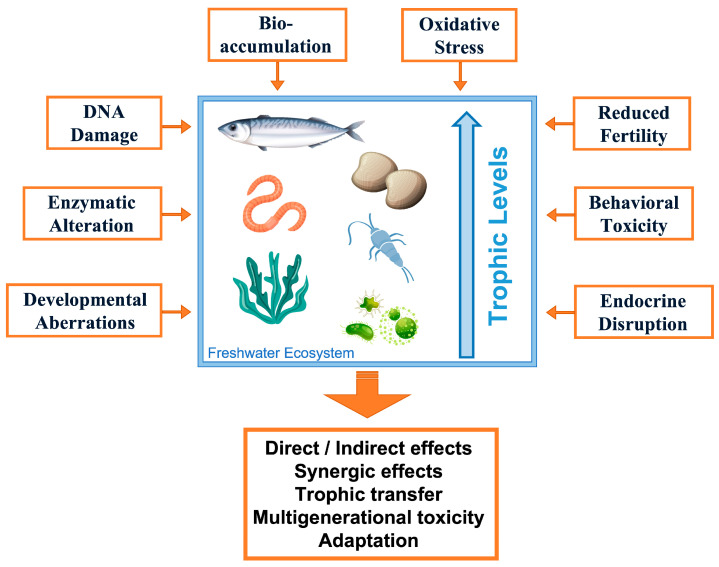
Schematic representation of the main anticancer drug effects on aquatic biota at the individual and ecosystem levels.

Hence, the chronic environmental release of these substances can impact reproduction, survival, and functioning in aquatic life. The calculated HC5 (the concentration at which 5% of species are potentially affected) for some anticancer drugs indicates they are hazardous contaminants for the environment [10]. As schematized in Figure 3, ACD effects to aquatic biota include bioaccumulation, oxidative stress, DNA damage, mitotic and developmental aberrations, enzymatic and behavioral alterations, reduced fertility, and endocrine disruption, which can determine further effects at individual and community levels [11]. However, ADCs’ fate in aquatic ecosystems remains largely unknown, and their potential impact on biota is poorly understood [10].

## 3. Current Limitations in ACDs Environmental Assessment

Despite increasing awareness of the environmental presence of ACDs, substantial limitations persist in the investigation of their aquatic behavior and effects.

***Scarcity of chronic and multigenerational toxicity studies across representative aquatic taxa***. While acute assays provide initial information, e.g., on lethality or growth inhibition, they fail to capture subtle, long-term, and heritable effects that may occur at environmentally relevant concentrations. ACDs are deliberately designed to interfere with fundamental and evolutionarily conserved cellular processes such as DNA synthesis, apoptosis, and signal transduction. As a result, even low-dose exposures may elicit non-threshold or delayed effects that standard ecotoxicological models fail to capture. Some of the most studied compounds, such as cyclophosphamide and ifosfamide, have been shown to induce chronic toxicity, genotoxicity, and reproductive impairments in fish, algae, and invertebrates [10]. These effects, particularly those involving DNA damage and cell-cycle disruption, raise concerns about potential transgenerational consequences and alterations at the population and community levels. However, systematic assessments encompassing multiple generations and ecological endpoints remain exceedingly limited, constraining the ecological relevance of current toxicity data.

***Trophic transfer and bioaccumulation***. Due to the relatively high lipophilicity and persistence of certain anticancer drugs and metabolites, these compounds can accumulate in the fatty tissues of aquatic organisms and be biomagnified along the food web. Although bioaccumulation factors have been estimated for a few cytostatics (e.g., tamoxifen [21] and platinum-based drugs [22]), empirical field data remain sparse. The possibility of trophic transfer not only heightens ecological risks but also raises potential human health implications through dietary exposure to contaminated fish or shellfish. Further studies are needed to quantify tissue-specific accumulation, trophic transfer efficiency, and depuration kinetics under realistic exposure scenarios.

***Synergistic or mixture toxicity***. In real aquatic environments, anticancer drugs seldom occur in isolation but rather coexist with a multitude of other pharmaceuticals, personal care products, metals, and organic pollutants. Such co-occurrence can result in additive or synergistic interactions that amplify toxicity beyond the sum of individual effects [23,24]. For example, D’Iglio et al. reported that exposing zebrafish larvae to the anticancer drugs Paclitaxel or Gemcitabine individually, both commonly used together in pancreatic cancer therapy, did not cause significant toxicity. In contrast, simultaneous exposure to both drugs at the same concentrations reduced the larvae’s survival and impaired their growth and development [25]. Experimental investigations into such mixture effects are still rare, and current risk assessment frameworks often overlook these interactions, assuming concentration addition or independent action modes that may not reflect environmental reality.

***Transformation and degradation products***. Anticancer drugs can undergo photolysis, hydrolysis, oxidation, or microbial transformation in natural waters and during wastewater treatment, producing metabolites or transformation products (TPs) that may retain, or even exceed, the toxicity of the parent compounds [3]. For instance, antimetabolites and alkylating agent photoproducts have shown persistent cytotoxicity and altered bioavailability [26]. However, the ecotoxicological profiles of most TPs remain largely uncharacterized, and their inclusion in monitoring and risk assessment frameworks is still exceptional. This gap is further complicated by analytical limitations in detecting and quantifying TPs at trace levels in complex environmental matrices.

## 4. Mesocosms to Advance Aquatic Ecotoxicology Testing of Anticancer Drugs

Addressing these gaps requires an integrative approach that combines multiple disciplines, e.g., across environmental chemistry, mechanistic ecotoxicology, molecular biology, to comprehensively elucidate the environmental fate, bioavailability, and biological impact of ACDs. One such approach is based on mesocosm experiments aiming at the reproduction and observation of the natural environment under controlled conditions.

**Figure 4:**
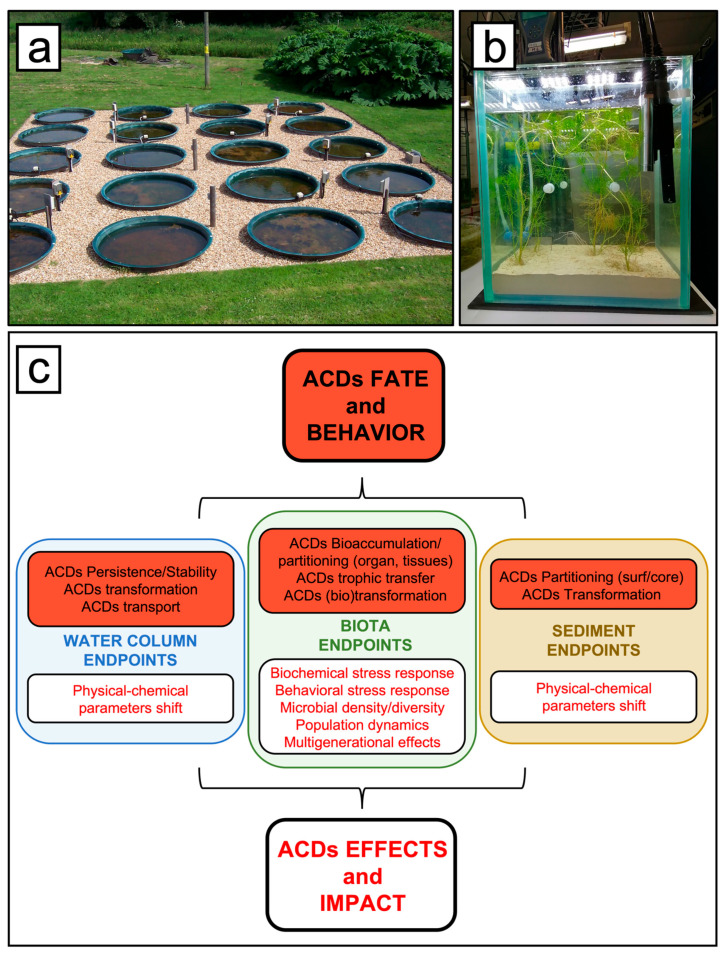
Representative (**a**) outdoor [27] and (**b**) indoor [28] freshwater mesocosm facilities mimicking lentic ecosystems. (**c**) A list of potential fate and effect endpoints simultaneously collected during the mesocosm experiments.

Mesocosms can be defined as enclosed, essentially self-sufficient environments that mimic a target ecosystem (Figure 4a,b). 

Their core components consist of a controlled experimental setting that effectively reproduces (i) natural abiotic conditions, for example, those related to the atmosphere, water, and sediment compartments, irradiation, and weather, and (ii) biotic parameters, such as the presence of microbial communities, macrophytes, and benthic and planktonic organisms spanning multiple trophic levels. Experimental designs can be tailored, for instance with regard to size, exposure time, and bio-physical-chemical parameters to address the research questions of interest, substantially offering a pragmatic middle ground between laboratory tests and field observations. Different experimental designs can mimic freshwater or saltwater ecosystem, comprehensive of the different environmental compartments, in either highly controlled indoor laboratory-scale platforms or in outdoor facilities trading a lower control on experimental parameters with more realistic environmental conditions [29]. 

Given the complex and long-term set-up and operation, such “high complexity/low throughput” experiments are not conceived for the screening/comparison of a large number of treatments but rather testing the most relevant ecological conditions. The contaminant dosing should reflect the concentrations actually measured in the ecosystem of interest and be designed to mimic the acute (pulse dosing) or chronic (press dosing) scenario under investigation. Yet, mesocosms should not be seen as alternative but rather complementary tools to standard “low-complexity/high-throughput” test, which can be used prior mesocosm, e.g., to define the best experimental conditions, or downstream, e.g., to deepen mechanistic aspects [30].

By simultaneously monitoring multiple fate and effect endpoints (Figure 4c), these experiments allow the characterization of the contaminants’ behavior (e.g., transport, transformation, distribution) as well as their direct and indirect impact on biota at both individual and community levels. Furthermore, they allow for the observation of mid- to long-term ecosystem trajectory, including response and recovery from contaminants and stressors [31,32].

Mesocosm experiments have been extensively used for the study of several classes of contaminants, from metals [33] to veterinary medicinal products [34], and particularly in the evaluation and regulation of pesticides [35,36]. More recently, they have been proposed to pursue the same goals concerning nano-pollution linked to nanomaterials [32], nano-enabled products [37], and micro-/nano-plastics [38]. When carefully designed, they can directly address most of the limitations in the assessment of ACDs in aquatic compartments and provide an effective platform for advancing ecotoxicology testing.

***Chronic and multigenerational effects of ACDs in representative taxa***. Mesocosm experiments are typically carried out in the months-to-year periods, allowing long-term observations. By sustaining planktonic and benthic populations (e.g., algae, invertebrates, and fish together) exposed to repeated low-level dosing, e.g., mimicking realistic excretion patterns or WWTP effluent pulses, organisms can be followed through life stages and across generations. This enables the measurement of reproductive endpoints, delayed effects, and transgenerational responses (e.g., fecundity, time to maturity, offspring quality) that short acute tests miss. This is relevant for ACDs, which often target conserved cellular machinery potentially causing cascade effects through populations and communities. Chronic, long-term exposures also allow monitoring hormesis and adaptation processes as well as ecosystem recovery after contamination [29,39]. Mesocosms also allow monitoring population dynamics and behavioral responses to contamination, sensitive indicators of noxious effects at concentrations below the lethal doses. For instance, aquatic macroinvertebrates displayed altered traits (locomotion, feeding) after exposure to a range of contaminants, including pharmaceuticals, pesticides, metals, or radioactive species [40,41,42]. Microbiota population density and composition is another sensitive indicator of ecosystem response. Overall, these metrics can be integrated with mechanistic indicators, such as molecular and cellular biomarkers addressing oxidative stress, to better describe adverse outcome pathways (AOPs) for these compounds.

***Trophic transfer and bioaccumulation of ACDs***. Because mesocosms are designed to include multiple trophic levels and natural feeding interactions, they are ideal for quantifying uptake, tissue distribution, and trophic transfer of lipophilic ACDs and metabolites. Tissues from primary producers, grazers, predators, and detritivores can be sampled over time to derive empirical bioaccumulation factors (BAFs) and trophic magnification factors (TMFs) under realistic diets and exposures, information that single-species lab assays cannot provide. For instance, recent mesocosm studies reported microplastic (MP) uptake in exposed zooplankton, altered wing morphology in chironomids, and consequent trophic transfer to odonate larvae at a higher trophic level [43]. Such processes can be correlated to direct and indirect effects on ecosystem (biotic and abiotic) parameters. As an example, Hua and Relyea reported direct, indirect, and trophic cascade effects of pesticides in freshwater, i.e., correlating the toxicity toward microcrustacean communities with effects on algal blooms, abiotic changes (water pH, dissolved O_2_), and macrobiota communities [44]. Mesocosms also allowed the identification of contaminant effects toward secondary consumers that were not evident in direct or simplified trophic chain exposure experiments [45].

***Mixture and synergistic toxicity of ACDs***. Mesocosms naturally accommodate complex contaminant mixtures (either ambient or experimentally spiked). By manipulating combinations, it is possible to observe emergent effects at the community and ecosystem level, such as food-web shifts, altered productivity, and species replacement, which are not predictable from single-compound additivity models. For instance, outdoor mesocosm studies showed how the impact of pesticides mixtures on amphibians could not be predicted on the basis of effects observed for individual pesticides [46]. In the context of ACDs, criteria for prioritizing the mesocosm testing of such mixtures should consider (i) the outcomes from standard ecotoxicity tests to prioritize the most hazardous drugs and (ii) the current therapeutic regimes and dosing, e.g., Paclitaxel and Gemcitabine cocktail concomitantly used in several cancer therapies, as well as (iii) environmental survey data informing on ACDs (co)occurrence and concentrations in the real aquatic compartments. Notably, mesocosms possibility for studying the synergistic/antagonistic effects from multiple stressors is not only confined to the straightforward exposure to a cocktail of a class of contaminants. For instance, previous studies highlighted the synergic effects of anthropogenic contaminants and nutrients on algal bloom in freshwater ecosystems [47]. More recently, several works investigated the combined impact of climate change stressors (e.g., heatwave) and contaminants such as pesticides [48] or nano-plastics [38].

***Transformation products and realistic fate processes of ACDs***. Sampling water, sediment, and biota over time allows the kinetic estimation of contaminant fate, including water persistence, transport, partitioning, and (bio)distribution within each natural compartment. In recent studies on engineered nanomaterials (ENMs), mesocosm helped clarify the realistic metal/ENM transformation and speciation, the preferential accumulation in specific compartments, and how such partitioning was altered when ENMs are incorporated in nano-enabled products [28,37,49,50]. Given the mid- to long-term exposures and reproduction of natural stressors, such as irradiation, varying redox conditions, and (micro)biotic activity, mesocosms are especially suited to kinetically investigate photochemical, hydrolytic, and biodegradation phenomena driving contaminants pathways that produce transformation products (TPs). For instance, wetland mesocosm allowed to qualitatively and quantitatively assess pesticides metabolites [51]. Correlating TP occurrence and kinetics to toxicity observations can help identifying whether TPs contribute to observed effects, which is critical for accurate risk assessment. Concerning ACDs, characterizing such behavior is fundamental to better assess, control, and regulate their presence in the environment.

***Reproducing realistic contamination scenarios of ACDs***. The reproduction of realistic contamination scenarios is a fundamental reason for implementing mesocosms. Future studies should prioritize the most exposed regional ecosystems identified in nature, for instance water basin receiving WWTP effluents. Relevant fate endpoints should evaluate the persistence, transport, bioavailability, and transformation of ACDs within these compartments. Effect endpoints should be carefully selected to estimate their impact, e.g., based on the mode-of-action of the ACD under investigation, and identify direct but also indirect effects of contamination. At these conditions, experimental outcomes may markedly differ from expectations based on laboratory tests. For instance, a recent study testing contaminated groundwater mixtures highlighted unexpectedly lower geno- and hepato-toxicity to fish than previously observed in laboratory tests, possibly due to a reduced bioavailability of the chemicals under investigation [52]. Other works reported toxicity thresholds much lower than those derived from standard single-species experiments [53].

In summary, well-planned mesocosm experiments can provide comprehensive and ecologically relevant datasets regarding ACD environmental fate as well as direct or indirect effects in a single experiment. Yet, their implementation for the environmental assessment of ACDs is surprisingly limited. To date, only one study directly addressed this topic focusing on the chronic effects of the chemotherapy drug cyclophosphamide (CP) in outdoor freshwater systems mimicking realistic worst-case scenario (total dose in the low µg/L ranges) for 3–4 months of exposure [53]. CP predominantly accumulated in the water column, consistent with its high water solubility and low K_ow_. However, dissipation times were faster than expected from laboratory tests and were ascribed to dissolved organic carbon content and the presence of biofilms. Regarding CPs’ effects, the study considered 18 taxa and reported no effects at the community-structure level but clear short-term adverse effects across cyanobacteria, rotifer, and microcrustacean populations. Here, NOEC was estimated to be <0.5 µg/L (the lowest concentration tested), much lower than previously reported values obtained from standard ecotoxicity tests. Overall, the research highlighted that the ecotoxicological risk associated with ACDs, such as CP, may be much higher than expected and displayed the added value of using realistic long-term, multispecies experimental strategies in the assessment of such risks.

Future research should adopt these strategies to expand current knowledge and produce comprehensive datasets, while also accounting for the limitations and design challenges of mesocosms. For instance, mesocosms are not meant to provide a comparison of a wide array of conditions (e.g., a range of contaminants concentrations), which must be selected in advance. Mesocosms can be implemented relatively easily in freshwater environments, especially when simulating small pond systems characterized by simple community structures. However, their effectiveness diminishes in more complex environments such as rivers, where they often overestimate contaminant effects. This is largely due to high water turnover and continuous flow, which hinder the maintenance of stable contaminant concentrations over extended periods. Marine environments are even more challenging, as long-term control of environmental parameters is difficult, and community structures are frequently oversimplified. Therefore, careful planning of experimental designs is essential, given the complexity of community structures and the difficulties associated with maintaining realistic environmental parameters over the long term. Biotic communities must be selected and introduced with attention to their ecological roles and interactions within the target ecosystem being mimicked, for example, to accurately represent trophic chains or food webs. These include both planktonic and benthic microbiota from endemic communities, considering their important role in driving contaminant fate [54]. At the same time, the endpoints to be assessed must be carefully chosen to detect direct and indirect effects across trophic levels, as well as to characterize ACD migration, transformation, and risk transmission. Table 1 provides categories of test organisms and their ecological roles, together with typical biomarkers and associated endpoints that can be monitored in mesocosm experiments.

Ultimately, because the experimental design inevitably reflects the expertise and interests of the operators, the absence of an interdisciplinary approach risks producing one-sided experiments that focus exclusively on either the fate of contaminants or their effects. Yet, the capacity to simultaneously generate comprehensive datasets on both hazard and exposure is one of the core rationales for implementing mesocosms. Therefore, experimental planning should involve researchers with diverse expertise—such as ecology, chemistry, biology, statistics, and informatics—and operational and analytical strategies should be defined from the outset to ensure that the resulting datasets are fully exploited.

## 5. Conclusions

Despite accumulating evidence of their environmental occurrence and biological effects, ACDs’ environmental behavior and impact remain largely unknown and require urgent actions. Recognizing these pharmaceuticals as priority contaminants and providing a comprehensive characterization of their environmental behavior and impact is essential to balancing human health benefits with ecological sustainability in the era of expanding cancer therapeutics. This perspective aimed to synthesize some current knowledge gaps in ACDs’ environmental fate and ecotoxicity assessment and emphasize the added value that testing at higher ecological complexity, such as mesocosm strategies, can provide.

Mesocosm approaches can help overcome some current limitations through (i) realistic exposure scenarios delivering comprehensive fate and effects datasets; (ii) chronic and multigenerational bioassays encompassing diverse aquatic species and trophic levels; (iii) systematic investigation of bioaccumulation and trophic transfer mechanisms; (iv) comprehensive mixture toxicity testing using environmentally realistic contaminant dosing and combinations; and (v) identification, quantification, and ecotoxicological characterization of transformation products.

The realistic exposure and hazard data collected can be fed to “higher-throughput” experiments, e.g., for mechanistic characterization of the processes observed, as well as fate models, hazard scoring schemes, and environmental risk assessment models. In turn, incorporating these priorities will enhance the ecological relevance of toxicity data, improve predictive risk models, and ultimately support the formulation of evidence-based policies for the safe management of anticancer drugs in aquatic environments.

## Figures and Tables

**Table 1:** A list of the categories of organisms to be incorporated into mesocosm experiments and their ecological roles, as well as related biotic indicators and endpoints that can be monitored.

Category Test Organisms	Ecological Role	Typical Biota Indicators	Typical Endpoints/Response to Exposure
Microbial community (e.g., heterotrophic bacteria, cyanobacteria, fungi)	– Nutrient cycling – Organic matter degradation – Microbial food web	– Biofilm biomass – Community composition/dynamics – Enzymatic activity – Resistance development (e.g., to antibiotics)	– Altered community structure and abundance – Altered metabolic profiles – Increased resistance
Primary producers (Autotrophs) (e.g., Phytoplankton, Periphyton, Macrophytes)	– Primary production—Oxygen generation – Nutrient uptake – Habitat structure, – 1st trophic level	– Chlorophyll content/photosynthetic efficiency – Biomass production – Mortality – Morphology (e.g., leaves, root) – Nutrient uptake efficiency, – ROS production/oxidative stress enzymes	– Photosynthesis inhibition – Growth inhibition – Impaired nutrient assimilation – Oxidative stress
Primary consumers (Invertebrates) (e.g., zooplankton, mollusks, insects)	– Nutrient cycling – Energy transfer in the food web – Ecosystem control (e.g., primary producers’ biomass) – 2nd trophic level	– Biomass – Community composition/dynamics – Biomarker (e.g., enzymatic activity, lipid peroxidation, ROS) – Behavioral markers – Reproductive parameters	– Behavioral alterations – Oxidative stress – Reproductive alteration – Altered community structure and abundance
Secondary consumers (Vertebrates, macroinvertebrate predators) (e.g., fish, insects, nymphs/larvae)	– Nutrient cycling, – Population control – 3rd trophic level	– Biomass – Community composition/dynamics – Biomarker (e.g., genotoxicity, enzymatic activity, DNA damage, ROS) – Behavioral markers – Reproductive parameters	– Behavioral alterations – Oxidative stress – Reproductive alteration – Altered community structure and abundance – Endocrine disruption – Altered gene expression

## Data Availability

Data sharing is not applicable.
